# Serum inhibin B concentration as a predictor of age at first menstruation in girls with idiopathic central precocious puberty

**DOI:** 10.1371/journal.pone.0205810

**Published:** 2018-12-14

**Authors:** Jérémie Tencer, Pierre Lemaire, Sylvie Brailly-Tabard, Raja Brauner

**Affiliations:** 1 Fondation Ophtalmologique Adolphe de Rothschild and Université Paris Descartes, Paris, France; 2 Univ. Grenoble Alpes, CNRS, Grenoble INP, G-SCOP, Grenoble, France; 3 Faculté de médecine Paris Sud, Université Paris Saclay and Assistance Publique-Hôpitaux de Paris, Hôpitaux Universitaires Paris Sud, CHU Bicêtre, Service de Génétique Moléculaire, Pharmacogénétique, Hormonologie, Le Kremlin-Bicêtre, France; University of Arkansas for Medical Sciences, UNITED STATES

## Abstract

**Objective:**

To compare the serum inhibin B, anti-Müllerian hormone (AMH) and leptin concentrations in girls with idiopathic central precocious puberty (CPP) to their concomitant characteristics and evaluate the capacity of each of these hormones to predict the age at first menstruation in those who were untreated and who completed their puberty.

**Methods:**

This single-center study included 94 girls selected from a cohort of 493 girls seen between 1981 and 2012 and diagnosed with idiopathic CPP for whom a remaining serum sample collected at the initial evaluation was available. Of these 25 were untreated and completed their puberty.

**Results- correlations at initial evaluation:**

In the whole cohort the inhibin B concentration displayed significant positive correlations with the age at the onset of puberty and at initial evaluation; bone age; breast Tanner stage; serum basal estradiol, luteinizing hormone (LH), follicle-stimulating hormone (FSH) and AMH concentrations, LH peak and LH/FSH peak ratio. The AMH concentration displayed a significant positive correlation with serum estradiol and a negative correlation with basal FSH concentration. The leptin concentration displayed significant positive correlations with the age at initial evaluation, bone age, and body mass index and a negative correlation with the FSH peak.

**Results- prediction of age at first menstruation:**

In 25 untreated girls, the inhibin B concentration was negatively correlated with the age at first menstruation (r = -0.61, p = 0.001) and the time between the onset of puberty and first menstruation (r = -0.59, p = 0.002). Inhibin B concentrations <30 pg/mL were associated with a time between the onset of puberty and first menstruation ≥3 years in 14/15 patients with a sensitivity of 0.67 and a specificity of 0.75. The age at first menstruation was estimated using a formula: min (11.15–0.510 LH/FSH peak ratio, 11.57–0.025 inhibin B)available at: http://www.kamick.org/lemaire/med/girls-cpp18.html.

**Conclusion:**

We established formulas based on the serum inhibin B concentrations and LH/FSH peak ratio at the initial evaluation, alone or in combination, to predict the age at first menstruation in girls with CPP. These formulas can assist with determining the indications for treatment in CPP.

## Introduction

Central precocious puberty (**CPP**) in girls is defined as the development of sexual characteristics before the age of 8 years due to premature activation of the hypothalamic-pituitary-ovarian axis. In girls, CPP is idiopathic in 80% of cases [1].

The occurrence of CPP in a given girl exposes her to a decrease in growth potential and age at first menstruation. The premature secretion of estradiol increases the growth rate and accelerates bone maturation, which can shorten the growing period, resulting in short adult height (**AH**). Treatment with a gonadotropin-releasing hormone (**GnRH**) analog blocks the pituitary-ovarian axis and thus estradiol secretion, thereby slowing bone age (**BA**) progression and preserving growth potential [2]. However, the effect of this treatment on AH varies, primarily because idiopathic CPP progression differs between slowly progressing forms and rapidly progressing forms [3]. It is difficult to determine whether to treat a given girl who has idiopathic CPP with GnRH analog since the reported height gain (AH-predicted AH at onset of treatment) varies from 0.3 to 9.8 cm [4]. The Consensus Conference Group has recommended that progressive pubertal development be documented for 3-6 months before starting treatment and that the responses to the GnRH test and estradiol assays should be monitored [2].

The second question posed by CPP is the decrease in age at first menstruation. It can be difficult for a girl aged less than 10 years to experience pubertal development, especially menstruation. To our knowledge, predictors of menarche after spontaneous puberty in CPP patients were not reported until our model [5]. In that study, the time between the onset of puberty and first menstruation was 3.6±1.5 years (0.9-7.8 years) and it was less than 2 years for only 9 (16%) girls. This time in untreated girls can be estimated with the formula 10.9–0.57 (LH/FSH peaks ratio). The formula is available at http://www.kamick.org/lemaire/med/girls-cpp15.html.

Inhibin B and anti-Müllerian hormone (**AMH**) are glycoproteins in the transforming growth factor-beta (TGF-β) family. Both are produced by the ovarian granulosa cells, inhibin B by small antral follicles and AMH by pre-antral follicles. Leptin is produced by fat cells under the control of the *ob* gene. Serum inhibin B, AMH and leptin concentrations vary during the puberty in girls. Studies measuring serum inhibin B or AMH concentrations before and after GnRH analog treatment in girls with CPP showed that the concentrations of both hormones decreased during treatment, suggesting that their secretion is linked to the activation of the hypothalamic-pituitary-ovarian axis [6,7].

O**ur objective** was to measure the serum inhibin B, AMH and leptin concentrations collected at the initial evaluation in girls with idiopathic CPP and to compare these concentrations with the clinical-biological presentation at the evaluation of the entire cohort. We also evaluated the capacity of each of these hormones concentrations to predict the age at first menstruation and AH in those untreated who completed their puberty and growth.

## Materials and methods

### Ethics statement

All clinical investigations were conducted according to the principals outlined in the Declaration of Helsinki. Written informed consent for the evaluation was obtained from the children’s parents and included in their hospital medical record. With the exception of routine patient care, no other interventions were performed as part of the study. The authors had no direct interaction with the patients enrolled in the study except during their medical follow-up, which was performed by R. Brauner. Patient information was anonymized by the medical secretary prior to analysis. The patients were selected from the cohort of a previous study [8] that was approved by the Ethical Review Committee (Comité de Protection des Personnes, Ile de France III) (Ref. CPP: AC 038), which states that “This study appears to be in accordance with scientific principles generally accepted and to ethical standards of research; this study was conducted according to French law and regulation”.

### Patients

We conducted a single-center study of 94 girls who were monitored for idiopathic CPP by a senior pediatric endocrinologist (R. Brauner) at a university pediatric hospital from June 1981 to July 2012 and for whom a remaining serum sample collected at the initial evaluation preserved at -22°C was available to measure the serum inhibin B, AMH and leptin concentrations. None of them had already undergone menstruation or reached the AH at inclusion.

**Fig 1:**
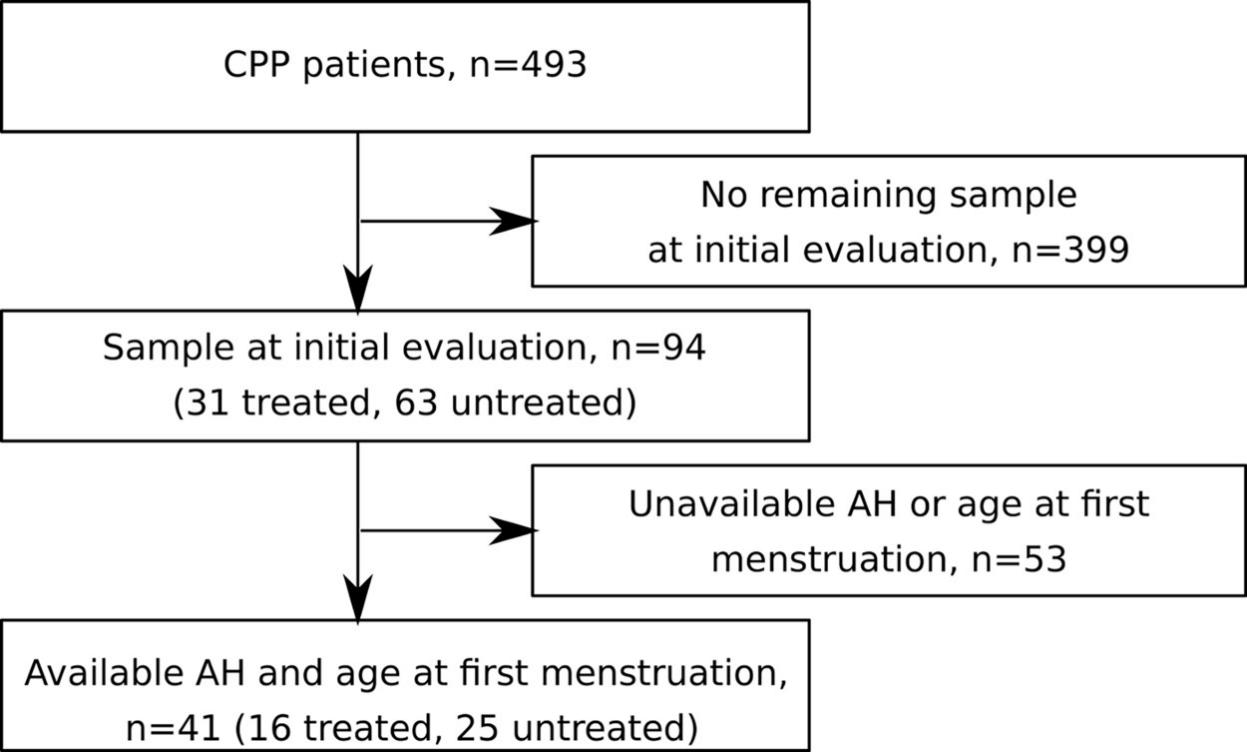
Flow chart of the inclusions of the girls with CPP.

The 94 girls included in the study were selected from a cohort of 493 girls with CPP [8] (Fig 1). At the initial evaluation, the characteristics of the 94 included girls were similar to those of the 399 girls without available samples with the exceptions of the Tanner stage of breast development, basal FSH concentration and LH peak, which were significantly lower in the included patients (Table 1).

**Table 1:** Comparison of the characteristics at initial evaluation of included and excluded girls with CPP.

	Included patients						Excluded patients			Mann-Whitney	Fisher
	Remarkable values	n	Mean±SD	Min	Max	%	n	Mean±SD	%	p	p
Age at onset, years		94	6.8±1.1	3.0	8.0		399	6.7±1.4		0.56	
	<6	19				20.2	69		17.3		
Age, years		93	7.5±1.4	3.5	9.5		399	7.6±1.5		0.27	
Tanner stage of breast		94	2.4±0.5	2.0	3.5		399	2.6±0.6			**0.0003**
Tanner stage of pubic hair		94	1.8±0.8	1.0	5.0		397	2.01±0.95			0.29
	≥2	57				60.6	250		63.0		
BMI, kg/m^2^		94	17.3±2.0	13.8	23.8		390	17.5±2.2		0.26	
BMI, SDS		94	1.4±1.5	-1.2	1.4	5.9	390	1.3±1.6		0.66	
	≥2	20				21.3	115		29.5		
Growth rate the year before, cm/year		90	7.5±2.1	5.0	13.0		354	7.8±2.2		0.45	
Growth rate the year before, SDS		90	1.8±1.8	-1.1	6.6						
	≥2	42				46.7					
Bone age advance, years		93	1.1±1.2	-1.3	5.5		385	1.3±1.3		0.15	
	≥2	27				29.0	118		30.5		
Uterus length, mm		61	34.0±8.1	20.0	58.0		209	37.0±8.9		0.06	
	≥35	27				44.3	122		58.4		
Basal LH concentration, IU/L		84	0.7±0.9	0.1	5.0		315	0.9±1.6		0.55	
Basal FSH concentration, IU/L		83	2.2±1.9	0.2	8.6		316	3.0±2.1		**0.0006**	
LH peak, IU/L		94	9.2±11.1	0.6	46.0		399	12.3±15.0		**0.018**	
FSH peak, IU/L		94	11.5±5.7	0.8	36.5		399	12.9±7.0		0.06	
LH/FSH peak ratio		94	0.9±1.3	0.09	7.63		399	10±1.1		0.07	
	≥0.66	34				36.2	190		47.6		
Estradiol, pg/mL		94	13.7±13.9	2.0	88.0		385	16.9±18.0		0.17	
	≥15	25				26.6	137		35.6		
Inhibin B, pg/mL		94	34.0±26.7	2.0	142.0						
AMH, pmol/L		89	19.6±14.0	0.0	73.8						
Leptin, ng/mL		94	8.6±6.7	0.4	33.1						

BMI: body mass index; LH: luteinizing hormone, FSH: follicle stimulating hormone, AMH: anti-Müllerian hormone, SD: standard deviation, min: minimum, max: maximum

The 94 included girls were selected from a cohort of 493 girls with CPP for whom a remaining serum sample collected at the initial evaluation was available.

The patients were assigned to one of two groups: treated patients (n = 31/94, 33.0%), who had been treated with a GnRH analog, and untreated patients (n = 63/94, 67.0%), who were followed without treatment. The criteria for undergoing treatment were a predicted AH <155 cm at the initial evaluation (n = 4), an LH/FSH peak ratio >0.66 (n = 21), and/or a serum estradiol concentration >15 pg/mL (n = 12). Patients were not treated (n = 63) because their predicted AH was ≥155 cm in all but 3 cases. These patients were monitored every 4 months until they reached an acceptable predicted AH for clinical and BA evaluations, if indicated.

The age at first menstruation and the AH (growth during the preceding year of less than 1 cm in a menstruating girl) are known for 41 girls (43.6%, 16 treated with the GnRH analog and 25 untreated). At initial evaluation, their characteristics were similar to those of the 53 without AH (p-values > 0.05 for all characteristics).

The 25 untreated girls (26.6% of the population) were analyzed to evaluate the capacity of serum inhibin B, AMH and leptin concentrations to predict the age at first menstruation and the AH.

### Methods

CPP was diagnosed based on the appearance of breast development before the age of 8 years in all patients, accompanied by the presence of pubic or axillary hair (n = 57/94, 60.6%), a growth rate greater than 2 standard deviations (**SDS**) the year before their initial evaluation (n = 42/90, 46.7%), and/or a BA more than 2 years greater than their chronological age (n = 27/93, 29.0%) (Table 1) [9]. We also considered CPP, but not premature thelarche, in girls for whom breast development at presentation was clinically isolated but was associated with a uterus length greater than 35 mm on an ultrasound (n = 27/61 evaluated, 44.3%) [10], an LH/FSH peak ratio greater than 0.66 after a GnRH test (n = 34/94, 36.2%) [11], and/or a serum estradiol concentration greater than 15 pg/mL (n = 25/94, 26.6%). According to these criteria, only 6 girls (6.4%) had isolated breast development at presentation, but their clinical picture became complete before 8 years of age; the ages at first menstruation were 10 and 11.5 years for the 2 patients for whom this information was available.

Organic intracranial lesions were excluded by neuroradiological evaluation in all but 12 (12.8%) patients, who had a family history of early puberty (n = 7), an age greater than 6 years at the onset of puberty (n = 11), a normal neurological evaluation (all), prepubertal serum estradiol concentrations (n = 9) and/or LH/FSH peak ratio <0.66 (n = 8).

The initial evaluation included the following data: the heights of each girl’s parents, ages at the onset of puberty (corresponding to the age at breast development), pubertal stage, height, weight, growth rate the year before the evaluation, BA, evaluation of the hypothalamic-pituitary-ovarian axis by measuring basal and GnRH-stimulated LH and FSH peaks and the serum estradiol concentration. A pelvic ultrasound was performed in 61 patients. For the GnRH stimulation test, we used Relefact (100 μg/m2), and the serum samples were collected 0, 30, 60 and 90 min after the injection. Serum LH, FSH and estradiol concentrations were measured with various radioimmunoassays during the study period. Each new assay for a given hormone was cross-correlated with the previous method to ensure that the results for a given parameter were comparable throughout the study period.

LH and FSH concentrations were measured using a two-site monoclonal immunoradiometric assay (LH-Coatria and FSH-Coatria; bioMerieux, SA, Marcy-l’Etoile, France). Estradiol was extracted with ether and measured using a radioimmunoassay (Estradiol-2; Sorin Biomedica, Antony, France). Serum inhibin B concentrations were measured with enzyme immunometric assays (Ansh Labs reagents, Webster, TX, US) [12]. The AMH concentrations in the same serum samples were determined using fully automated immunoassays – Access 2 (Beckman Coulter Company, Marseille, France) [13]. The lower limits of detection were 3 pg/mL and 0.14 pmol/L for inhibin B and AMH, respectively. Leptin concentrations were measured using radioimmunoassay (Millipore Corporation, Billerica, MA, US), which had a lower limit of detection of 0.45 ng/mL [14].

Height, growth rate and BMI (weight in kg/height in m^2^) were expressed as SDS for chronological age [15,16]. The pubertal stage was rated according to the method reported by *Marshall and Tanner* [17]. BA was assessed by R. Brauner for all patients according to the *Greulich and Pyle* method [18]. The target height was calculated based on parental heights [19].

### Analysis

Pearson’s correlation coefficients were computed for all variables. Linear regression analyses were conducted for two outcomes: AH and age at first menstruation. In both cases, we restricted ourselves to models using only variables that were determined at the initial evaluation, and several non-linear transformations of the variables were systematically carried out (inverse, quadratic or logarithmic transformations). Relevant subsets of variables or transformations of variables were computed using the "step" method of GNU R [20]. The quality of the models was further challenged specifically for predictions using the following standard cross-validation procedure [21]: 80% of the dataset was randomly and uniformly selected as training data to compute a model, and the remaining 20% was used to evaluate this model. This procedure was repeated 1000 times, and the average performance of those 1000 iterations was retained. Models for which performances collapsed in cross-validation were considered non-reliable and were discarded.

Data are expressed as the means±SD.

## Results

### Characteristics at the initial evaluation (Table 1)

Of the 94 girls, 49 (52.1%) had 1 or 2 pubertal signs, 39 (41.5%) had 3 to 6 pubertal signs associated with breast development, and 6 (6.4%) had isolated breast development (See Patients). Twenty (21.3%) patients were obese (BMI ≥2 SD). The obese group was similar to the non-obese group except for the presence of pubic hair (p = 0.048), advanced bone age (p = 0.047) and serum leptin concentrations (15.2±7.5 ng/mL vs 6.6±5.1 ng/mL, p<0.0001) that were significantly higher in the obese group.

### Correlations in the whole population (Table 2)

**Table 2:** Correlations between serum inhibin B, AMH, and leptin concentrations and the characteristics at initial evaluation of girls with CPP.

	Inhibin B				AMH				Leptin			
	n	r	95CI	p	n	r	95CI	p	n	r	95CI	p
Age at onset, years	94	**0.25**	[0.05; 0.43]	**0.01**	89	-0.08	[-0.29; 0.13]	0.44	94	0.17	[-0.04; 0.36]	0.10
Age at initial evaluation, years	93	**0.30**	[0.11; 0.48]	**0.003**	88	-0.11	[-0.31; 0.10]	0.30	93	**0.24**	[0.04; 0.42]	**0.02**
Bone age, years	93	**0.26**	[0.06; 0.44]	**0.01**	88	0.06	[-0.15; 0.27]	0.57	93	**0.23**	[0.03; 0.42]	**0.03**
Tanner stage of breast development	94	**0.49**	[0.32; 0.63]	**<0.0001**	89	0.16	[-0.05; 0.35]	0.14	94	**-0.03**	[-0.23; 0.18]	**0.79**
BMI, kg/m^2^	94	**0.04**	[-0.16; 0.24]	**0.67**	89	0.05	[-0.16; 0.25]	0.65	94	**0.65**	[0.51; 0.75]	**<0.0001**
Estradiol, pg/mL	94	**0.28**	[0.08; 0.45]	**0.007**	89	**0.22**	[0.01; 0.41]	**0.04**	94	-0.07	[-0.27; 0.13]	0.49
Basal LH concentration, IU/L	84	**0.27**	[0.06; 0.46]	**0.01**	79	-0.06	[-0.28; 0.16]	0.61	84	-0.01	[-0.22; 0.20]	0.93
Basal FSH concentration, IU/L	83	**0.30**	[0.09; 0.49]	**0.005**	78	**-0.34**	[-0.53; -0.13]	**0.002**	83	-0.20	[-0.40; 0.01]	0.06
LH peak, IU/L	94	**0.39**	[0.20; 0.55]	**0.0001**	89	-0.07	[-0.28; 0.14]	0.50	94	-0.14	[-0.33; 0.07]	0.18
FSH peak, IU/L	94	-0.03	[-0.23; 0.17]	0.78	89	-0.20	[-0.39; 0.01]	0.07	94	**-0.29**	[-0.46; -0.09]	**0.005**
LH/FSH peak ratio	94	**0.33**	[0.13; 0.50]	**0.001**	89	-0.03	[-0.24; 0.18]	0.77	94	-0.09	[-0.28; 0.12]	0.41
Inhibin B, pg/mL					89	**0.35**	[0.15; 0.52]	**0.0009**	94	0.02	[-0.18; 0.22]	0.84
AMH, pmol/L	89	**0.35**	[0.15; 0.52]	**0.0009**					89	0.12	[-0.09; 0.32]	0.28
Leptin, ng/mL	94	0.02	[-0.18; 0.22]	0.84	89	0.12	[-0.09; 0.32]	0.28				

BMI: body mass index; LH: luteinizing hormone, FSH: follicle stimulating hormone, AMH: anti-Müllerian hormone, 95CI: 95% confidence interval

The serum inhibin B concentration displayed significant positive correlations with the age at the onset of puberty and at the initial evaluation; BA; Tanner stage of breast development; number of signs of puberty associated with breast development (n = 94, r = 0.37, 95% CI [0.18; 0.53], p = 0.0002); serum basal estradiol, LH, FSH and AMH concentrations; LH peak and LH/FSH peak ratio (Table 2).

The serum AMH concentrations displayed significant positive correlations with the number of signs of puberty associated with breast development (n = 89, r = 0.23, 95% CI [0.02; 0.42], p = 0.03) and serum estradiol concentration and a negative correlation with basal FSH concentration.

The serum leptin concentrations displayed significant positive correlations with the age at initial evaluation, BA and BMI and a negative correlation with the FSH peak.

### AH and age at first menstruation (Table 3)

**Table 3:** Correlations between serum inhibin B, AMH, and leptin concentrations and the characteristics after the end of growth and puberty of girls with CPP.

		Inhibin B				AMH				Leptin			
		n	r	95CI	p	n	r	95CI	p	n	r	95CI	p
Untreated	Age at 1st menstruation, years	25	**-0.61**	[-0.81; -0.28]	**0.001**	24	-0.40	[-0.69; 0,00]	0.05	25	-0.07	[-0.45; 0.34]	0.75
	Time between onset of puberty and 1st menstruation, years	25	**-0.59**	[-0.80; -0.25]	**0.002**	24	-0.27	[-0.61; 0.15]	0.20	25	-0.31	[-0.63; 0.09]	0.13
	Adult height, cm	25	-0.005	[-0.40; 0.39]	0.98	24	-0.08	[-0.47; 0.33]	0.70	25	0.18	[-0.24; 0.53]	0.40
	Difference between target and adult heights, cm	23	-0.12	[-0.50; 0.31]	0.60	22	0.03	[-0.45; 0.40]	0.89	23	-0.32	[-0.65; 0.10]	0.13
Treated	Age at 1st menstruation, years	16	-0.03	[-0.52;0.47]	0.90	16	0.09	[-0.42;0.56]	0.73	16	0.09	[-0.43;0.56]	0.74
	Time between onset of puberty and 1st menstruation, years	16	-0.28	[-0.6;0.25]	0.29	16	0.51	[0.02;0.80]	0.05	16	0.02	[-0.48;0.51]	0.94
	Adult height, cm	16	0.27	[-0.26;0.68]	0.31	16	-0.16	[-0.61;0.36]	0.54	16	0.31	[-0.22;0.70][	0.25
	Difference between target and adult heights, cm	12	-0.12	[-0.65;0.48]	0.70	12	0.19	[-0.43;0.69]	0.55	12	-0.52	[-0.84;0.08]	0.08

BMI: body mass index; LH: luteinizing hormone, FSH: follicle stimulating hormone, AMH: anti-Müllerian hormone, 95CI: 95% confidence interval

In the untreated girls, the mean AH was 164.1±6.2 cm, with 2 patients presenting an AH less than 155 cm. AH and the difference between the target and adult heights (-1.6±4.4 cm) did not correlate with inhibin B, AMH and/or leptin concentrations (Table 3). None of the new models (using inhibin B, AMH and/or leptin concentrations) was significantly better than the baseline model at predicting AH [5].

**Fig 2:**
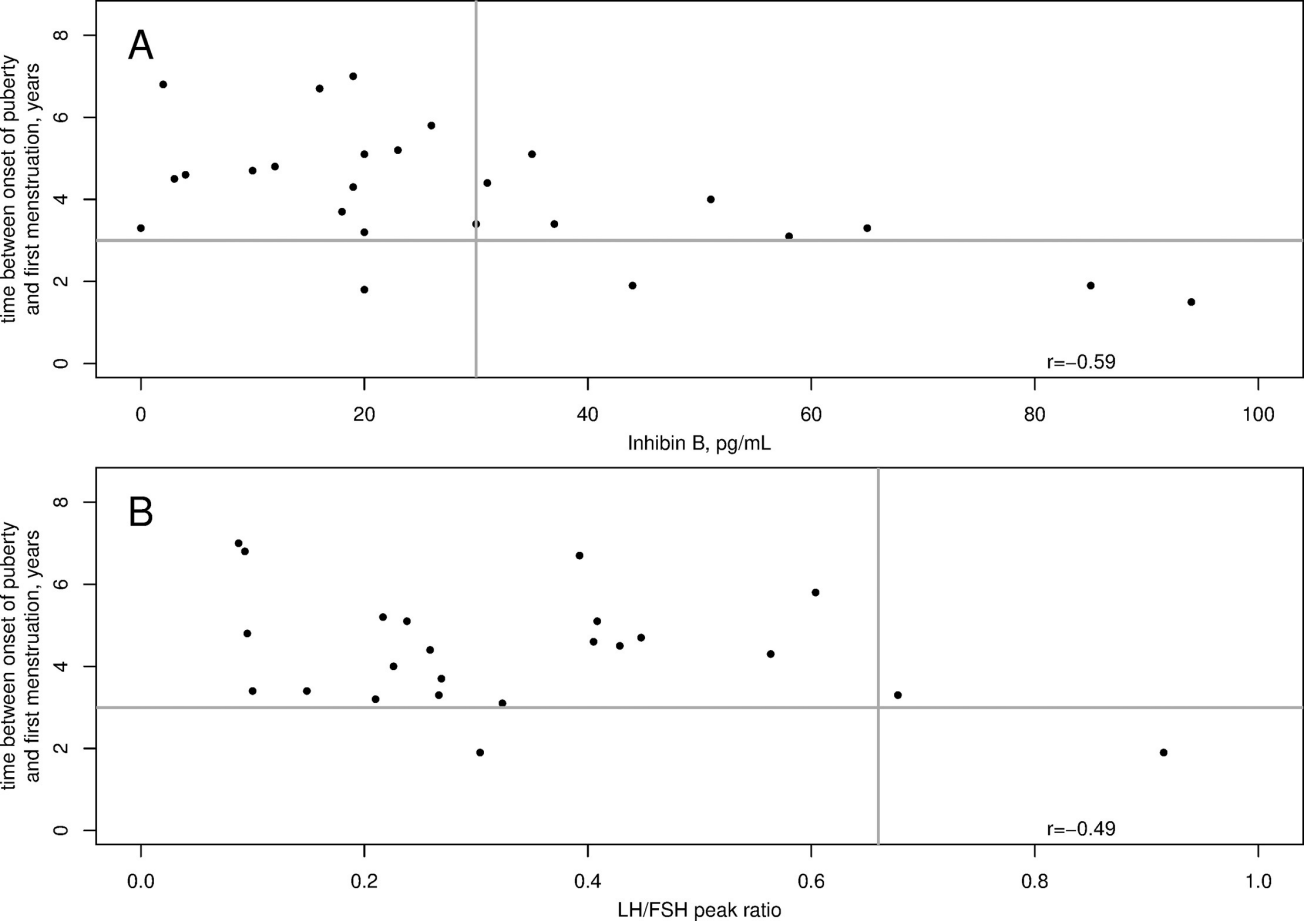
**Time between onset of puberty and first menstruation vs the inhibin B concentration (A) or LH/FSH peak ratio (B) in 25 girls with untreated CPP.** Each dot is one girl. Data from two patients are not included in B, as their LH/FSH peak ratios were 5.68 and 2.9; their times between onset of puberty and first menstruation were 1.5 and 1.8 years, respectively.

The mean age at first menstruation was 10.8±1.0 years. The first menstruation occurred before 10 years of age in 5 girls (20.0%), between 10 and 11 years in 8 girls (32.0%) and between 11 and 13 years in 12 girls (48.0%). The time between the onset of puberty and first menstruation was 4.1±1.5 years; it was less than 2 years for only 4 girls. The age at first menstruation and the time interval between the onset of puberty and first menstruation were negatively correlated with the serum inhibin B concentration but not with the serum concentrations of AMH or leptin at the initial evaluation. The age at first menstruation and the time between the onset of puberty and first menstruation also correlated negatively with the LH peak (p = 0.015) and with the LH/FSH peak ratio (p = 0.011). Serum inhibin B concentrations <30 pg/mL were associated with a time interval between the onset of puberty and first menstruation ≥3 years in 14/15 patients, with a sensitivity of 0.67 and specificity of 0.75 (Fig 2).

In the treated girls, the mean AH (160.3±5.7 cm), the difference between the target and adult heights (0.2±7.6 cm), the age at first menstruation (11.9±0.9 years) and the time interval between the onset of puberty and first menstruation (5.3±1.5 years) did not correlate with inhibin B, AMH and/or leptin concentrations nor with the LH/FSH peak ratio.

**Fig 3:**
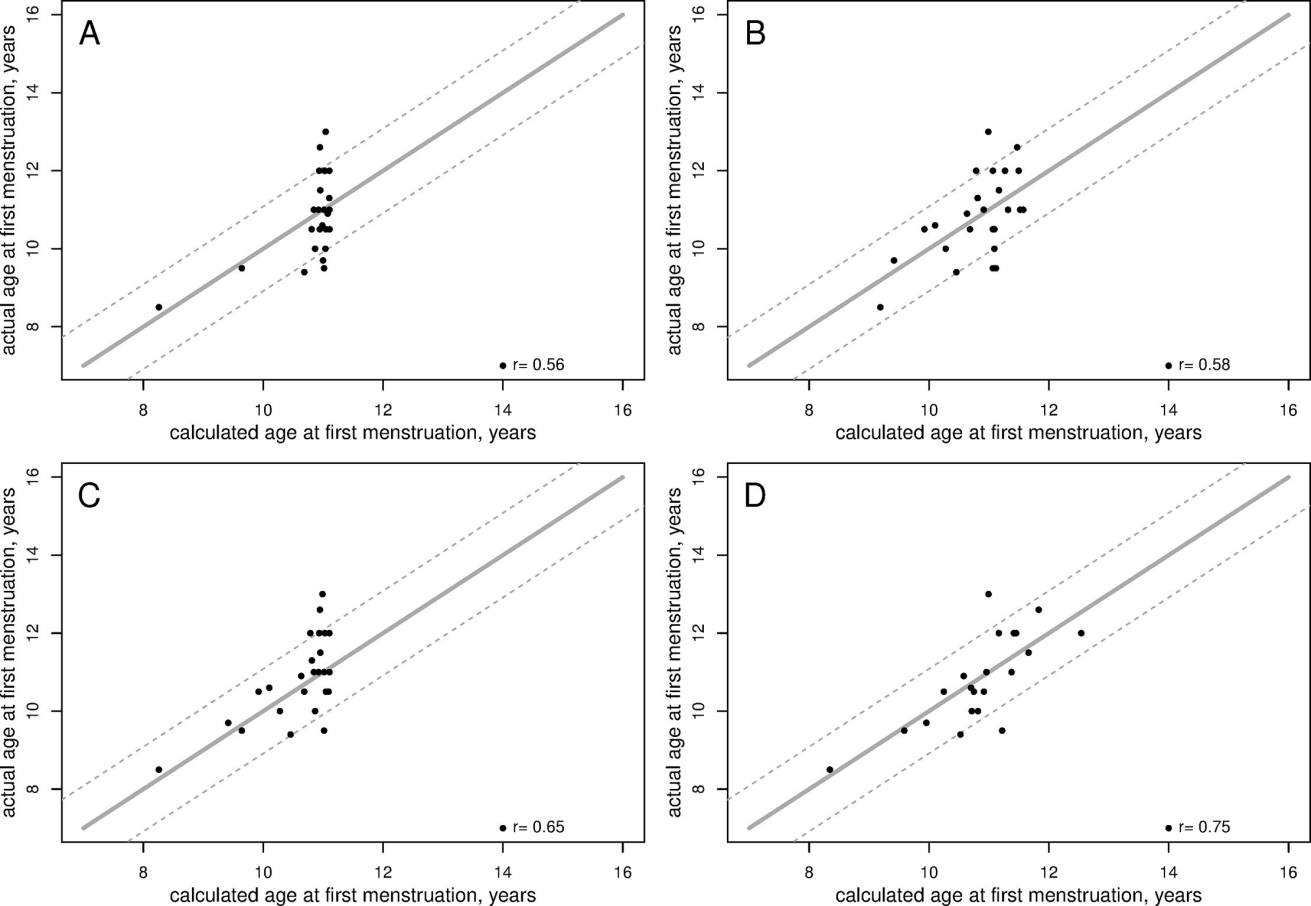
Correlations between the actual and calculated ages at first menstruation in 25 girls with untreated CPP. Ages are calculated using (A) serum inhibin B concentrations (formula 1); or (B) LH/FSH peak ratio (formula 2); (C) the lowest of the two predictions (formula 3); or (D) the non-linear model (formula 4). Each dot is one girl. Dot-lines correspond to ±1 SD (1.09 years). Models are available online at: http://www.kamick.org/lemaire/med/girls-cpp18.html.

The age at first menstruation (M1) was estimated using the following formulas based on the serum inhibin B concentration or the LH/FSH peak ratio (Fig 3A and 3B):

M1=11.15−0.510[LH/FSHpeaksratio] (1)

M1=11.57–0.025[InhibinBconcentration] (2)

These two formulas had similar overall performances. They achieved r = 0.56 (p = 0.004) and r = 0.58 (p = 0.003) for the whole dataset and r = 0.43 and r = 0.50 upon cross-validation, respectively, with an average absolute error of 0.80 year; each model overestimated the actual interval by more than one year for 4 girls (16.0%) and underestimated it by more than 1 year for 3 girls (12.0%). It is notable that the model based on both variables was discarded because it did not perform better than any of the two above formulas. However, the use of the lower of the two predictions led to better results (Fig 3C):

$$M1=min\;\left\{\begin{array}{c} 11.15–0.510[LH/FSH\;peaksratio]\\ 11.57–0.025[InhibinBconcentration] \end{array}\right\}$$ (3)

This combined model achieved r = 0.65 (p = 0.0004) for the whole dataset and r = 0.57 upon cross-validation respectively, with an average absolute error of 0.77 years; this model overestimated the actual interval by more than one year for 2 girls (8.0%) and underestimated it by more than 1 year for 4 girls (16.0%).

Using additional variables (TH and AMH) and non-linear transformations, a more complex model can be proposed (Fig 3D):

$$\begin{array}{c} M1=10.62–0.38[LH/FSH\;peaksratio]+0.44[TH(SD)]\\ –\frac{0.75}{\left[AMHconcentration\right]}+\frac{9.84}{1+[InhibinBconcentration]} \end{array}$$ (4)

This model achieved r = 0.76 (p<0.0001) for the whole dataset and r = 0.65 upon cross-validation, but with an average absolute error of 1.68 years; it overestimated the actual interval by more than one year for 2 girls (9.1%) and underestimated it by more than 1 year for 1 girl (4.5%). Other parameters (e.g., BMI) did not lead to the finding of better, more reliable models.

All formulas are available online at http://www.kamick.org/lemaire/med/girls-cpp18.html.

## Discussion

We established formulas based on the serum inhibin B concentration and LH/FSH peak ratio at the initial evaluation, alone or in combination, to predict the age at first menstruation in girls with CPP.

### Serum inhibin B concentration in girls with CPP

In healthy girls, the serum inhibin B concentration is low until the age of 8 years, increases from the beginning of puberty though Tanner stage 3, and then decreases [22]. To the best of our knowledge, four studies have analyzed the contribution of the serum inhibin B concentration to the care of girls with CPP. Sehested et al. evaluated 42 girls with CPP and found that their median inhibin B level (80 pg/mL) was greater than those of age-matched controls (21 pg/mL), but the value was appropriate for their Tanner stage [7]. Crofton et al. evaluated 11 girls with premature thelarche and found that their inhibin B and FSH levels were greater than those of age-matched controls (p<0.01 and 0.05, respectively) and similar to those of 13 girls with CPP and of normal pubertal Tanner stage-matched controls [23]. De Filippo et al. used inhibin B concentrations to differentiate patients with non-progressive CPP (n = 31) from those with progressive CPP (n = 31); the latter group displayed an LH peak >7 IU/L and the presence of additional criteria related to breast development than patients with the non-progressive type [24]. They found inhibin B concentrations of 13.1 and 29.1 pg/mL (p<0.001), respectively, with patients with the non-progressive forms having similar concentrations as their prepubertal controls of similar ages (11.3 pg/mL). Our population included patients of the same age as the De Filippo study, and we used the same assay. However, we included patients with both non-progressive and progressive CPP according to the classification reported by De Filippo et al. In that study, the combination of a serum inhibin B concentration of 20 pg/mL and a basal LH level of ≤0.2 IU/L results in 98% sensitivity and specificity to differentiate the non-progressive form from the progressive form. Chen et al. evaluated inhibin B concentrations in girls with CPP, which was defined as an LH peak greater than 5 IU/L and the presence of additional criteria related to breast development. The progressive form was defined as the progression of at least two criteria (Tanner stage, bone age advance, or growth velocity) for 6 months [25]. They observed lower plasma inhibin B concentrations in patients with slow-progressive CPP (n = 28, 33.63 pg/mL) than in patients with progressive CPP (n = 55, 60.8 pg/mL), but the difference was not significant. The authors obtained more discriminating results using AMH level (see below) and the combination of AMH and inhibin B levels, with an optimum inhibin B concentration cut-off 30.12 pg/mL.

### Serum AMH concentration in girls with CPP

Two longitudinal studies describe peripubertal changes in AMH secretion. Its levels increased by 17% starting 3 years prior to pubertal onset and then decreased by 30% during the 2 years after the onset of puberty [26,27]. In a study of girls with CPP, Hagen et al. used the same assay as the present study and reported a median plasma AMH concentration of 20.3 pmol/L in 13 girls with CPP and 2 girls with advanced puberty before GnRH analog treatment, values similar to those of age-matched controls (23 pmol/L) and Tanner stage-matched controls (19 pmol/L) [6]. Chen et al. reported mean plasma AMH concentrations of 20.1 and 38.4 pmol/L (p = 0.0047) in patients with slow-progressive and progressive forms of CPP, respectively [25]. The AMH concentration had an 80% sensitivity and an 89.3% specificity for differentiating slow-progressing to progressive CPP, with a 26.9 pmol/L cut-off.

### Serum leptin concentration in girls with CPP

Serum leptin concentrations also increase significantly during puberty in girls [28,29]. The role of leptin in the initiation of puberty remains unclear. Most girls with idiopathic CPP have a higher BMI than same-aged girls in the general population [1]. Their serum leptin concentrations are correlated with their BMI and with the weight increase during the year preceding the evaluation, but not with the LH/FSH peak ratio.

We observed a mean leptin concentration of 8.6±6.7 ng/mL, with a median of 6.8 ng/mL. Larmore et al. used the same assay to examine 12 older non-obese girls with CPP who had a higher mean BMI than our population and reported a mean plasma concentration of 7.7 ng/mL, which was lower than that of obese age-matched prepubertal girls (n = 12) (18.6 ng/mL, p<0.004) but similar to that of non-obese age-matched prepubertal girls (n = 12, 5.5 ng/mL) and normal pubertal girls Tanner stage-matched (n = 12, 4.8 ng/mL) [30]. Yoo et al. used a different assay in 30 non-obese girls with CPP and reported a mean leptin concentration of 5.0 ng/mL before GnRH analog treatment [31].

### Correlations at initial evaluation

In the present study, the serum inhibin B concentration displayed significant positive correlations with almost all concomitant characteristics of the girls with CPP. In a longitudinal study of 6 girls with normal puberty, Crofton et al. showed that plasma inhibin B concentrations had strong positive correlations with serum estradiol (r = 0.79, p = 0.001) and FSH (r = 0.80, p = 0.001) concentrations [32]. Sehested et al. showed that plasma inhibin B concentration was significantly positively correlated with the plasma estradiol concentration during Tanner stages 1 and 2; with the basal LH concentration in all stages except Tanner stage 3; and with the FSH concentration in all stages except 2 and 3 [22]. In girls with CPP, Crofton et al. reported a significant positive correlation between serum inhibin B and FSH concentrations (r = 0.55, p = 0.05) [23].

In the present study, the serum AMH concentration displayed significant positive correlations with number of signs of puberty associated with breast development and the serum concentrations of estradiol and inhibin B. It negatively correlated with the basal FSH concentration. Hagen et al. reported a significant negative correlation between AMH and FSH concentrations in 224 prepubertal girls (r = -0.31, p<0.001) [26]. They also observed this correlation in 13 girls with CPP (r = -0.856, p<0.001).

This study is the first to report a significant negative correlation between the leptin level and the FSH peak. Yoo et al. evaluated estradiol, peak and basal LH and FSH levels before and 6 months after GnRH analog treatment but did not observe significant correlations [31].

### Prediction formula

In a previous study, we used a mathematical model to predict the age at first menstruation using only the LH/FSH peak ratio [5]. In the present study, the inhibin B concentration and the LH/FSH peak ratio displayed negative correlations with the time interval between the onset of puberty and first menstruation. Our preferred method for predicting the age at first menstruation is to use both formulas and to choose the lower value (Fig 3C), because this method achieves a good compromise between simplicity of use and quality of prediction. More accurate predictions can be achieved using formula 4 (Fig 3D), but at the cost of determining AMH concentrations.

### Limitations

This study has limitations. It was a retrospective study. The exclusion of 399 (80%) girls due to the absence of a remaining biological sample may have introduced bias [5]. We postulate that the similarities in between the characteristics of the excluded girls and of those who were included with regard to the variables analyzed (except for the Tanner stage of breast development, LH peak and basal FSH concentration) limit this bias.

The age at first menstruation and the AH are available for only 41 (44%) of the patients. This may have introduce bias. We postulate that the similarities of their characteristics at the initial evaluation with those of the 53 patients without available AH limits this bias. The formula for predicting the age at first menstruation using the inhibin B concentration was established based on only 25 untreated girls, but its results were consistent with our reported formula [5] when we replace the inhibin B concentration with the LH/FSH peak ratio.

The absence of a correlation between the inhibin B concentration and AH may be due to the limited number of girls in the cohort who achieved their AH after spontaneous growth. A tool that can predict AH in patients with CPP can be used in clinical decisions about whether treatment is indicated. In a recent paper on internal and European validation of formulas to predict the AH, we included 34 girls (of these 6 had samples available and were included in the present study) we followed in the same conditions [33]. These girls did not differ from our original population used to establish the formula, except for the percentage of treated girls, which was significantly greater in [8] than in the validation study.

The major limitation of our study is the lack of validation of the formulae on a separate population.

## Conclusions

Our results suggest that a serum inhibin B concentration <30 pg/mL is associated with a time interval ≥3 years between the onset of puberty and first menstruation in almost all girls with untreated idiopathic CPP. The inhibin B concentration displays a predictive power similar to that of the LH/FSH peak ratio in models that were established to predict the age at first menstruation in this population. Thus, the measurement of serum inhibin B concentration may supplement and, in some cases, replace the GnRH test. This finding is of particular interest in terms of the cost of medical care for girls with idiopathic CPP. However, this result must be confirmed in studies using internal and external validation methods.

## Supporting information

S1 Appendix STROBE checklist. (PDF)

S1 File The complete data. This is a CSV file containing all the anonymized data used in this article and ready to be imported in any spreadsheet or data-analysis software.
(CSV)
